# Proton Therapy in Uveal Melanoma

**DOI:** 10.3390/cancers16203497

**Published:** 2024-10-16

**Authors:** Adrian Wai Chan, Haibo Lin, Irini Yacoub, Arpit M. Chhabra, J. Isabelle Choi, Charles B. Simone

**Affiliations:** 1 Department of Radiation Oncology, Sunnybrook Health Sciences Centre, The University of Toronto, Toronto, ON M4N 3M5, Canada; 2 New York Proton Center, New York, NY 10035, USA; 3 Department of Radiation Oncology, Memorial Sloan Kettering Cancer Center, New York, NY 10065, USA

**Keywords:** uveal melanoma, proton therapy, ocular tumor, plaque brachytherapy, reirradiation, enucleation

## Abstract

**Background/Objectives:** Uveal melanoma is the most common primary intraocular malignancy in adults. Treatment options for localized, early-stage disease include enucleation, brachytherapy, and proton beam therapy. This review aims to evaluate the role of proton therapy in the definitive management of uveal melanoma, focusing on its physics, radiobiology, treatment techniques, and associated outcomes. **Methods:** This narrative review synthesizes current literature on proton therapy for uveal melanoma, emphasizing case selection, treatment efficacy, and side effects. **Results:** Proton therapy offers significant advantages for thicker uveal melanomas (over 8 mm) due to its unique physical properties, including a rapid dose fall-off that protects critical structures like the retina and optic nerve. Proton therapy may have benefits in tumor control for ocular melanomas given its increased relative biological effectiveness relative to photon therapy for these typically more radioresistant melanomas. Proton therapy may also hold special value for uveal melanomas in close proximity to the optic nerve, as patients are at high risk of visual toxicities with brachytherapy. The review discusses the efficacy of proton therapy across small, medium, and large tumors, along with strategies for improving patient survival through combined systemic therapy. Additionally, the potential of ocular reirradiation with proton therapy is addressed. **Conclusions:** Proton therapy is an effective treatment for uveal melanoma. It offers advantages over brachytherapy for large tumors, tumors that are close to the optic nerve or insertion of extra-ocular muscles.

## Simple Summary

Uveal melanoma is a common eye malignancy and is managed by surgery, brachytherapy or external beam radiation for early-stage disease. Proton therapy is a newer form of radiation therapy and is especially suited to treat large uveal melanoma because of its unique physical characteristics. This review describes the physics, radiobiology, treatment techniques, and clinical outcomes when using proton therapy to treat uveal melanoma, with a focus on choosing between brachytherapy and proton therapy.

## 1. Background

Uveal melanoma is the most common primary intraocular malignancy in adults and accounts for 3–5% of all melanomas. It most commonly arises from melanocyte in the choroid (85–90%), and less commonly arises in the iris (3–5%) or ciliary body (5–8%) [1]. The incidence is much higher in non-Hispanic Whites (6 per million people), whereas it is lower in Hispanic (1.67 per million), Asian (0.38 per million), and Black (0.31 per million) populations [2]. Uveal melanoma can be classified by its size in several ways, one of which is based on its thickness, into small (≤3 mm), medium (>3–8 mm), and large (>8 mm) [3].

Treatment of early-stage uveal melanoma includes enucleation, brachytherapy, and proton beam therapy [4]. For metastatic uveal melanoma, therapies such as site-directed chemotherapy (i.e., intra-arterial liver chemotherapy), regional, metastasis-directed or even selective internal radiation therapy, chemotherapy, immunotherapy, and targeted therapy are all standard treatment approaches [5].

This review article describes the use of proton therapy for the definitive management of localized uveal melanoma, with a particular focus on physics, radiobiology, treatment technique, case selection, clinical outcomes, and side effects.

## 2. Radiation Therapy for Melanoma

Melanoma is considered a radioresistant tumor type; therefore, early-stage melanoma in non-ocular sites, such as cutaneous melanoma, is typically treated with wide local excision [6]. In the case of uveal melanoma, surgical resection via enucleation of the eye causes permanent vision loss and can result in major social, mental, and emotional distress. As such, radiation therapy is more commonly used in uveal melanoma compared with cutaneous melanoma.

Ocular structures, such as the retina and optic nerve, may be damaged by radiotherapy. Typical dose constraints for the retina and optic nerve are maximum doses of 45 Gy and 54 Gy, respectively [7]. Doses above these levels can cause permanent vision loss. However, the standard dose required to control macroscopic melanoma is 66–70 Gy [8]. This creates a dilemma: how can we deliver adequate doses to the uveal melanoma, while keeping doses to the retina, optic nerve, and other ocular structures as low as possible?

Brachytherapy, which involves the suturing of a plaque containing a radioisotope such as ruthenium-06 or iodine-125 to the sclera of the eyeball, has long been used in the definitive treatment of uveal melanoma [3,9]. The short range of penetration of the beta radiation emitted by these radioisotopes enables the delivery of a high dose to the tumor and a relatively lower dose to the retina and other ocular structures. The dose fall-off by inverse square law is sharpest in the first 5–7 mm [10]. Larger uveal melanomas thicker than 8 mm will derive less benefit from brachytherapy, as the retina or optic nerve will receive a dose similar to that delivered to the tumor. Traditional photon radiotherapy has a more gradual dose fall-off compared with brachytherapy and has more limited use in the treatment of uveal melanoma.

## 3. Proton Physics

**Figure 1:**
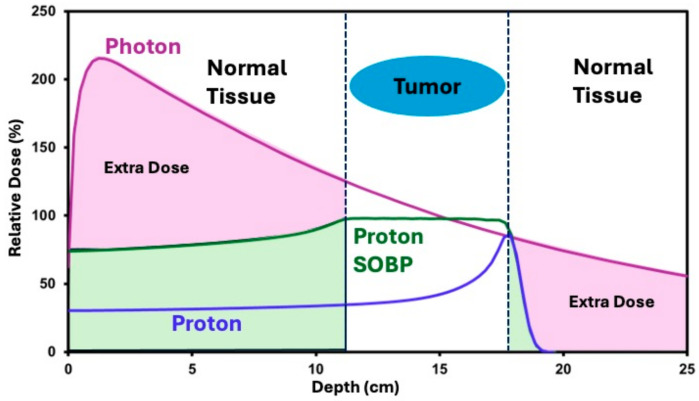
Difference in dose fall-off between photon and proton. SOBP = spread-out Bragg peak.

Protons are charged particles that interact with atomic electrons and nuclei through coulomb interaction. As the protons enter and slow down in the human body, the radiation dose deposited increases steeply in a short distance at the end of the proton’s range, followed by a sharp fall-off beyond the peak as the protons come to rest [11]. This peak in the dose before the rapid fall-off is called the Bragg peak. When the radiation target or tumor is placed at the depth of the Bragg peak, there can be significant sparing of critical structures beyond the target or tumor due to zero exit dose (see Figure 1) [12]. Because of this unique physical property of protons, proton therapy is well suited to treat uveal melanoma, particularly large or juxtapapillary tumors that are not good candidates for brachytherapy [3].

Beyond their rapid dose fall-off, protons react differently to tumors compared with photons. While photons primarily interact with human tissue or tumors by Compton scattering, protons predominantly deposit their dose through multiple coulomb scattering with atomic electrons. Along with the higher relative biological effectiveness (RBE) of proton therapy [13], the linear energy transfer (LET), which is defined as the average energy deposited in a medium by a charged particle over a given distance, is higher for protons near the Bragg peak than photons [11]. It has been suggested that the radioresistance of melanoma might be overcome by a higher LET radiation, in addition to a higher physical dose [14]. This forms the theoretical biological basis for the use of proton therapy in uveal melanoma.

## 4. Uveal Melanoma Proton Treatment Technique

After initial investigations and standard imaging evaluation including computer tomography (CT) and magnetic resonance imaging (MRI) of the orbit, patients requiring proton treatment undergo surgical placement of tantalum marker rings, which are inserted at the edge of the tumor on the sclera. As the border of the uveal melanoma may not be clearly seen in the cross-sectional images for radiotherapy planning, the tantalum marker rings can aid tumor delineation. In addition, during radiation treatments, these markers can be visualized with daily imaging guidance to ensure consistent stepping up of the eyeball, including KV imaging and cone beam CT, which are increasingly available in proton facilities [15].

Patients are immobilized in a head mask to ensure a reproducible setup. Eye gaze is typically placed in a specific position, with the direction of gaze often selected according to the target location and the patient’s vision capability. Commonly, the gaze is fixed contralateral to the target, often bringing the tumor to a more superficial location. Such eye gaze fixation allows for both enhanced setup reproducibility and the potential to decrease irradiation doses to organs at risk.

Gross disease is delineated, and a margin of 2–3 mm is typically added to the tumor to account for microscopic disease, setup uncertainties, and beam uncertainties. An additional circumferential margin of 1 mm may be added to account for setup uncertainty at the treatment team’s discretion [16,17]. The usual prescribed dose range includes 56–60 Gy (RBE) in 4 fractions and 50–70 Gy (RBE) in 5 fractions [16,18].

**Figure 2:**
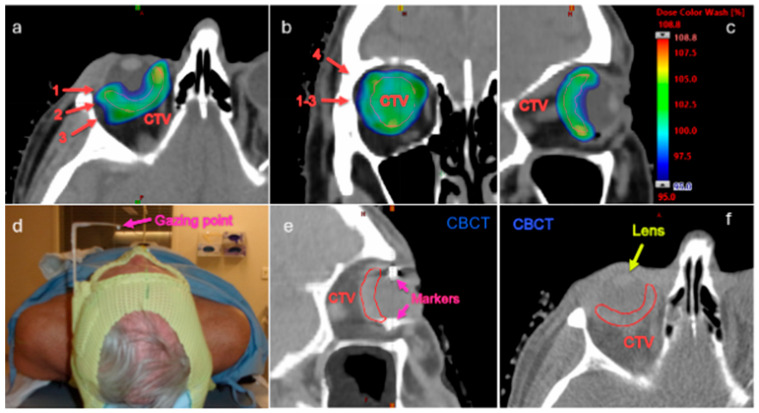
Gantry-based PBS proton treatment plan for uveal melanoma patient (**a**–**c**). The patient was planed with four fields, including one non-coplanar beam (red arrows). The patient was treated in a supine position with a gaze fixation device (**d**). Imaging guidance includes CBCT alignment to the surrounding bones, tantalum markers, and visible anatomies (**e**,**f**). CTV = clinical target volume. CBCT = cone beam computed tomography.

Proton therapy for uveal melanoma is typically delivered in treatment facilities with a dedicated “eyeline” treatment room, which affords the delivery of proton therapy through a single anterior beam with a small aperture in a sitting position [19]. Treatment planning is typically facilitated by the use of an idealized eye model. However, pencil beam scanning (PBS) is increasingly being delivered for non-ocular targets in proton centers [20], and our institution, along with others, has very recently begun using proton therapy to treat uveal melanoma with a regular PBS gantry in the absence of a dedicated eyeline. Figure 2 presents a gantry-based ocular treatment case, showing the 3D dose optimized with four beams (a–c), the immobilization and gazing fixation devices (d), and the imaging guidance with daily CBCT for marker and anatomy alignments (e,f).

## 5. Efficacy of Proton Treatment in Large Choroidal Tumors (>8 mm)

Papakostas et al. reported a series of large choroidal metastases treated by proton therapy in a tertiary center in Massachusetts [21]. A total of 336 tumors were treated, with a median tumor height of 8.7 mm (range: 2.0–17.1 mm), and 61% of the tumors were within 2 disc diameters from the optic disc. These tumors were less suitable for brachytherapy because of their large size and proximity to the optic disc. The median follow-up times for ocular outcome and survival were 3 and 7 years, respectively. The rate of tumor control at 10 years was 87.5%, and tumors that were at least 18 mm in largest basal diameter were more likely to recur than smaller tumors, with a relative risk of 3.5. Despite the excellent local control, the 10-year overall survival rate was 39.3%, as 48.5% of patients in this cohort died of metastatic uveal carcinoma within 10 years. This is similar to the 10-year mortality rate of 40% from melanoma metastasis n the enucleation arm of the Collaborative Ocular Melanoma Study [22].

For the ocular outcomes, the eye retention rate was 77.4% at 5 years and 70.4% at 10 years [21]. The authors did not report the reasons for enucleations. Despite the high rate of eye retention, visual acuity deteriorated steadily after treatment. At 1 year after treatment, visual acuity was 20/200 or better in 48.6% of patients treated with proton therapy. This proportion dropped to 22.6% at 3 years and only 8.7% at 10 years. In patients whose tumors were located more than 2 disc diameters from the optic nerve, one-third could retain at least 20/200 visual acuity 10 years after treatment. It should be noted that the reliability of ocular outcomes data may be limited by the relatively short median follow-up time of 3 years. Of note, however, the use of Kaplan–Meier estimates of vision loss may overestimate actual vision loss, as it does not account for patients who recover their vision after initial loss, such as the common scenario of patients who develop cataract and subsequently undergo lens replacement.

Other studies on the use of proton therapy in large uveal melanoma showed similar results [23,24]. Bensoussan et al. reported the outcomes of proton beam therapy for 492 choroidal melanoma patients with AJCC 7th Edition T3-T4 disease and a mean tumor diameter and thickness of 14.9 mm and 8.8 mm, respectively [23]. At a median follow-up of 61.9 months, the local control at 5 years was 94%. The overall survival rates at 10 years for T3 and T4 patients were 52% and 40%, respectively. By 15 years, 30% of T3 and 43% of T4 patients developed distant metastases. Risk factors for the development of metastases included juxtapapillary location, extrascleral extension, and ciliary body extension. Among the 19.5% of patients who underwent enucleation after proton therapy, only 32.3% experienced a local relapse. Other reasons for enucleation included neovascular glaucoma (30.2%), unreliable surveillance (24.8%), and phthisis (12.5%). The authors did not elaborate further on whether “unreliable surveillance” meant that the patient could not adhere to the surveillance protocol or if the follow-up imaging results were deemed unreliable [23].

In summary, these studies have consistently demonstrated that proton therapy can achieve excellent and durable local control (>90%) and high rates of eye retention (approximately 80%), but with poor visual acuity in the long term, as more than 80% of patients have a visual acuity of less than 20/200 by 10 years. Most enucleations after treatment were performed for treatment complications instead of local recurrences. Importantly, the very high local control did not obviate the development of distant metastases, which occurs in 30–50% of patients. This resulted in a 10-year overall survival rate of approximately only 50% [25].

## 6. Potential Role of Combining Proton Radiotherapy with Systemic Treatment

The study results discussed above lead to the question of how we can improve treatment outcomes for patients with uveal melanomas. Dose escalation or other interventions to boost the local control are unlikely to improve survival in the majority of patients, as the local control rate following proton treatment is already excellent—approximately 90%—with proton therapy doses. Clinical studies have shown that escalation of dose from 50 Gy to 70 Gy did not improve the local control [26,27]. Even upfront enucleation, which has excellent local control, has a 10-year mortality rate from melanoma of approximately 40% [22]. This is likely because most patients with uveal carcinoma have already developed early micro-metastases long before the treatment of their primary ocular tumors [28]. Among patients who had distant metastasis, the liver (93%), lung (24%), and bone (16%) were the most common sites of metastases [29]. In spite of this, staging workup, including positron emission tomography (PET), computer tomography (CT), and abdominal ultrasound only identified metastases in 2.8% of patients in a cohort of uveal melanoma over 4 mm in thickness [30].

To target such micro-metastases that are not detectable by imaging, systemic treatment is the most viable option. In order to achieve the maximal chance of cure, systemic therapy may optimally be administered around the time of local treatment when the tumor burden is still low. Uveal and cutaneous melanomas display extreme differences in their genetic alterations and biological behaviors; therefore, the standard treatment for metastatic cutaneous melanoma may not be equally effective for uveal carcinoma [31].

There are ongoing efforts to identify effective adjuvant treatments for uveal melanoma. Tebentafusp is a bispecific gp100 peptide-HLA-directed CD3 T cell engager that was shown in a phase 3 randomized controlled trial to prolong overall survival (21.6 months in the tebantafusp group versus 16.9 months in the control group) in patients who were positive for HLA-A*02:01 and had unresectable or metastatic uveal melanomas [32]. A retrospective study showed that radiotherapy may augment the response to tebentafusp in patients with metastatic uveal melanoma, presumably through radiotherapy-induced immunogenic cell death [33,34]. The ATOM trial is a randomized trial with an estimated start date of October 2024 that compares adjuvant tebentafusp to placebo after surgery or radiotherapy in patients with HLA-A*02:01 positive primary non-metastatic high-risk uveal melanoma [35].

The accrual of the ATOM trial is estimated to take 8 years, completing in late 2032. This is likely due to the rarity of uveal carcinoma, and is comparable to the 11.5 years that were needed to enroll patients into the Collaborative Ocular Melanoma Study (COMS) [29]. Furthermore, the frequency of the HLA-A*02:01 allele in the population varied between 11.9% in African or African American patients to 27.1% in individuals of European descent. There continues to be an unmet need for new systemic approaches in patients who do not carry the HLA-A*02:01 allele. Previous trials evaluating the efficacy of adjuvant treatment, such as fotemustine or crizotinib in high-risk uveal melanoma, failed to show significant benefits [36,37]. Until an effective systemic treatment for early-stage uveal melanoma is identified, the long-term survival after proton radiotherapy will likely remain poor, even among patients with durable local control. Another potential future direction may be the better selection of treatment based on morphological or functional imaging. Radiomics analysis in pre-treatment PET-CT has been shown to predict melanoma outcome and its response to target therapy and immunotherapy, though it remains to be determined whether it can help guide the selection of local treatment including proton therapy [38].

## 7. Efficacy of Proton Treatment in Small (≤3 mm) or Medium (3–8 mm) Uveal Melanoma

Although proton therapy has mostly been used for large-sized uveal carcinoma, in part because brachytherapy is less effective in these patients, proton therapy has also been used to treat medium size uveal melanoma. Caujolle et al. analyzed the results of 886 patients who had uveal melanoma with a median tumor diameter of 15.7 mm and a median thickness of 5.7 mm [39]. In the entire cohort, the local control, metastasis-free survival, and overall survival rates at 10 years were 92.1%, 76.4%, and 64.1%, respectively. The eye retention rate in the entire cohort at 10 years was 87.3%. These outcomes were compared favorably with those having larger uveal melanomas [21,23,24]. Overall survival was reported according to the T stage. The 10-year overall survival for patients with T1, T2, T3, and T4 tumors were 86%, 78%, 43%, and 41%, respectively.

While local control and overall survival in T1 and T2 tumors treated with proton therapy are relatively good, proton therapy may not be required for all patients. Similar outcomes can generally be achieved by plaque brachytherapy. One study showed that the uveal melanoma-related mortality after plaque brachytherapy in small- or medium-sized tumors (median thickness: 5.2 mm) was 28% at 10 years [40]. This is comparable to outcomes achieved with proton therapy [41]. Given the similar outcomes between proton therapy and brachytherapy in small- or medium-sized tumors, treatment modality selection should take into account both patient and tumor factors. Geospatial access to proton therapy may also be a determinant in the therapeutic approach recommended for these patients [42].

## 8. Choosing between Brachytherapy and Proton Therapy

### 8.1. Tumor Thickness

The dose of brachytherapy falls off rapidly with distance, following the inverse square law [43]. This has two implications. First, uveal melanoma usually has a base that is attached to the choroid and sclera. The other side of the tumor is referred to as the apex, and the distance between the base and apex depends on the tumor thickness. Because of the rapid dose fall-off in radiotherapy, maintaining sufficient dose throughout a thick tumor can be difficult. A meta-analysis involving 3,913 patients treated primarily with 106 Ru plaque brachytherapy for uveal melanoma showed that a higher tumor thickness was associated with a higher risk of local failure [44].

Second, if brachytherapy is used for thick tumors, an adequate dose needs to be prescribed to the tumor apex to maintain local control. The sclera and choroid, which are much closer to the brachytherapy source, can receive a dose several times higher than that delivered to the tumor apex. In one report, one patient with a tumor thickness of 12 mm insisted on brachytherapy, which necessitated a very high dose of 1449 Gy to the tumor base and 2340 Gy to the scleral surface [45]. Such a high dose to the sclera significantly increases the risk of scleral necrosis, which can occur in up to 33% of cases after brachytherapy for uveal melanoma [46]. A higher dose to the choroid and retina also increases the risk of permanent decline in visual acuity. In a retrospective review of 113 consecutive ocular melanoma patients with follow-up of visual acuity, a tumor thickness of 5.1 mm or greater and AJCC category T3 were both associated with a greater decline in visual acuity [47].

Proton therapy delivers a more homogenous dose throughout the entire tumor compared with brachytherapy, and therefore, may be favored in patients with thick tumors to avoid the high risk of scleral necrosis.

### 8.2. Tumor Location

Another important factor to consider when choosing between proton therapy and brachytherapy is the tumor location. Uveal melanomas that are close to the optic nerve head are difficult to treat with brachytherapy for two reasons. Brachytherapy involves suturing a radioactive plaque to the sclera where the tumor is located. In order to deliver an adequate dose to the entire tumor and any microscopic disease, the tumor base plus a margin (e.g., 2 mm) should be covered by the prescribed brachytherapy dose [48]. This means that the edge of the plaque will need to extend beyond the rim of the tumor. If the optic nerve head is located immediately adjacent to the tumor, the optic nerve head can create an obstacle to properly suturing the radioactive plaque to the tumor and surrounding sclera. This may be partially circumvented by the use of a slotted radioactive plaque, which has a notch that allows the entire optic nerve to pass through [10].

In addition to the difficulty in placing the radioactive plaque, the proximity of the optic nerve to the tumor can increase the risk of optic neuropathy. As discussed earlier, the radiation dose from the brachytherapy source has a very rapid fall-off, and any normal tissues, including the optic nerve, that are very close to the source can receive a dose significantly higher than the prescribed dose. This can lead to optic atrophy and irreversible vision loss [49]. Because of the reasons discussed above, some patients with uveal melanomas close to the optic nerve may be more safely treated by proton radiotherapy.

Anteriorly located tumors, such as iris and conjunctival melanomas, may also benefit from treatment by proton therapy rather than plaque brachytherapy. The unique anatomy of the iris makes it difficult to attach the brachytherapy plaque to it [50].

### 8.3. Quality of Life

One of the issues that is less often adequately addressed is patient preference and quality of life. Brachytherapy involves two general anesthesia sessions, first for the suturing and second for the removal of the radioactive plaque, although the use of local anesthesia has been explored as well [51]. While a lead shielding eye patch is usually sufficient to decrease the irradiation emitted from the radioactive plaque, in many practices, patients are admitted to hospital for the duration of the brachytherapy for pain control and to obviate potential concerns regarding delays in plaque removal due to patient-specific causes (e.g., failure to return for removal and radiation safety issues). Comparatively, proton therapy does not involve the placement or removal of a radioactive plaque under anesthesia. Patients typically do not have to be admitted for treatment. The entire course can be completed within 1–2 weeks, although surgical placement of tantalum marker rings at the tumor border under anesthesia to aid in treatment planning is still required [52].

Gollrad et al. conducted a longitudinal assessment of the quality of life of patients who received proton therapy for uveal carcinoma, using standardized questionnaires such as the EORTC QLQ-C30 and EORTC QLQ-OPT30. They found that there was a short-term decline in quality of life, most likely related to the surgery to place the markers before radiation therapy [53]. However, the questionnaires were administered only up to 3 months after treatment, so this study could not capture the long-term quality of life of patients who received proton treatment. Other studies reporting the long-term outcomes of proton therapy most often focused on visual acuity and survival, and quality of life longitudinal data have rarely been assessed [21].

In order to help clinicians and patients decide between proton therapy and brachytherapy, future research should prospectively assess and compare both the short-term and long-term quality of life of patients who are treated with these two modalities. When interpreting the results of these studies, clinicians should bear in mind that a patient’s preference likely depends on several other factors, including their willingness to be admitted to hospital, fitness for repeated anesthesia, and ability to commute to hospitals for fractionated proton therapy.

**Figure 3:**
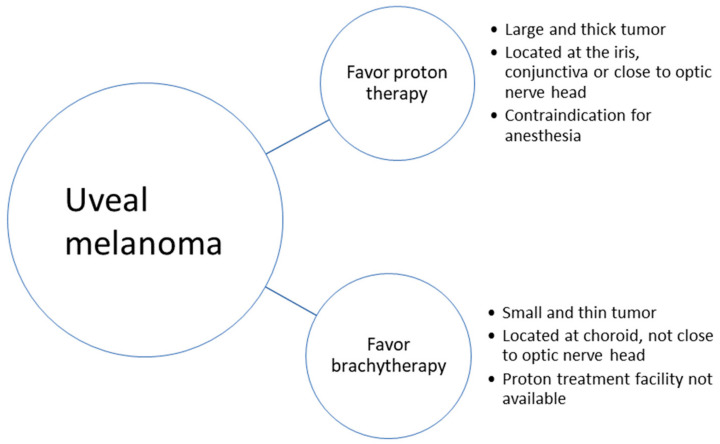
Considerations when choosing between proton therapy and brachytherapy.

Figure 3 gives a summary of the relevant factors when choosing between proton therapy and brachytherapy.

## 9. Side Effects

Potential side effects after proton treatment for uveal melanoma include radiation retinopathy, optic neuropathy, and neovascular glaucoma, all of which may also occur after brachytherapy (see Table 1 for complications of proton treatment) [49]. Marinkovic et al. reported on the reasons for enucleation after proton therapy for 103 patients with uveal melanoma with a median thickness of 8.4 mm. Fifteen patients (14.4%) had enucleations for treatment toxicities after proton therapy, including neovascular glaucoma (*n* = 3), retinal detachment +/− rubeosis (*n* = 3), neovascular glaucoma and retinal detachment (*n* = 3), and vitreous hemorrhage with blindness (*n* = 2). These data highlight the significance of treatment complications. Neovascular glaucoma deserves special mention, as it is a major complication necessitating enucleation [49].

Neovascular glaucoma occurs as a result of iris ischemia from radiation therapy, causing elevated intraocular pressure and permanent visual field loss from glaucoma [54]. A qualitative systematic review showed that neovascular glaucoma could be more common in proton therapy compared with brachytherapy, with an incidence of 23–38.1% after proton therapy and up to 17% after brachytherapy [55]. It should be noted, however, that the mean tumor thickness has been shown to be predictive of the incidence of neovascular glaucoma [56], and as discussed above, proton treatment is generally favored when treating uveal melanoma > 8 mm. This systematic review did not analyze patient-level data or correct for the effect of tumor size on the incidence of neovascular glaucoma, so its conclusion should be interpreted with caution.

Another retrospective review comparing proton treatment and radioactive iodine 125 implant did not find any difference in the incidence of neovascular glaucoma, although brachytherapy was associated with higher rates of diplopia, cataract, and maculopathy [57]. The extra-ocular muscles are sometimes temporarily disinserted when positioning the implant, especially if the tumor is juxtaposing the muscle insertion on the globe. In this study, the need for muscle detachment during brachytherapy was highly correlated to the incidence of diplopia. This may suggest that patients whose tumors are located immediately adjacent to the insertion of extra-ocular muscles may be best managed by proton therapy.

## 10. Follow-Up after Proton Treatment

Serial orbital MRI is usually recommended for the follow-up of uveal melanoma after the completion of treatment, as it can detect a reduction in tumor size before it becomes apparent in ultrasound [58]. Notably, however, one study showed that at a mean of 8 months after proton therapy, nearly two-thirds (64.7%) of patients had a reduction in tumor volume, but patients might need to be monitored for up to 32 months to see a final response to proton therapy [59].

## 11. Reirradiation

In patients with local-only recurrences to prior radiation therapy, proton therapy may be uniquely suited to attempt to reduce the risks of toxicities in the setting of reirradiation [60]. Proton reirradiation for ocular tumors has been delivered following prior proton therapy, photon therapy, and brachytherapy. A systematic review of proton reirradiation for ocular and central nervous system tumors reports on six studies [61], the largest of which involved a cohort of 31 patients with recurrent uveal melanoma. These patients were, initially, most typically treated to 70 Gy RBE, and then they were retreated, at a median time of 36 months later, most typically again to 70 Gy RBE. The mean follow-up time after the second treatment was 50 months (range: 6–164 months). At 5 years, local recurrence was only 31%, and more than half (55%) of patients were able to retain their eye. Overall, proton reirradiation was well tolerated, with expected cataract formation in 39% and visual acuity declines over time in several patients, but no major ocular complications were reported [62].

## 12. Conclusions and Future Directions

Proton therapy is an effective treatment for uveal melanoma. It offers advantages over brachytherapy for large tumors, tumors that are close to the optic nerve or insertion of extra-ocular muscles, and for patients unable to tolerate anesthesia. Despite the high local control rate achieved with definitive management, the 10-year rate of distant metastasis of up to 50% represents a remaining significant challenge in the treatment of uveal melanoma. Future research is needed to assess whether combining systemic therapy and proton therapy may eradicate distant micro-metastases and the primary tumor, in a complementary and/or synergistic approach, thus leading to more favorable long-term survival, and whether radiomic analysis may help select the appropriate treatment. Further assessments comparing the impact of brachytherapy and proton therapy on short-term and long-term quality of life for patients with uveal melanomas are indicated.

## Figures and Tables

**Table 1:** Complications after proton treatment for uveal melanoma.

Anterior Chamber and Lens	Vitreous Chamber	Retina and Optic Nerve
Cataract	Vitreous hemorrhage	Retinal detachment
Neovascular glaucoma		Radiation maculopathy
Rubeosis iridis		Optic neuropathy
